# Childhood Abuse and Caregiving for Perpetrating Parents: Impacts on Adult Child Well-Being

**DOI:** 10.1093/geroni/igab046.1241

**Published:** 2021-12-17

**Authors:** Jaime Goldberg, Jooyoung Kong, Sara Moorman

**Affiliations:** 1 University of Wisconsin-Madison, Madison, Wisconsin, United States; 2 Boston College, Chestnut Hill, Massachusetts, United States

## Abstract

Combining the stress process model of caregiving and life course perspective, this study examined the long-term influences of childhood abuse on perpetrating parent-adult child relationships and adult child well-being in the context of caregiving. Using a sample of family caregivers from the Wisconsin Longitudinal Study (969 caregivers of mothers; 280 caregivers of fathers), we investigated whether contact frequency and emotional closeness with an abusive parent mediate the longitudinal effects of parental childhood abuse on adult child caregivers’ depressive symptoms and the moderating effects of self-acceptance and mastery on this mediational association. Key findings indicate that maternal childhood abuse may negatively affect emotional closeness between an adult child caregiver and perpetrating mother (b = -0.24, p < .001). This could lead the adult child caregiver to experience increased depressive symptoms (b = 0.02, p < .05). Although the mediation paths for the effect of maternal childhood abuse on depressive symptoms via emotional closeness with mothers did not differ by caregivers’ level of psychological resources, we found that psychological resources significantly moderated the association between maternal childhood abuse and depressive symptoms (b = -0.08, p < .05). Further research may explore this phenomenon in light of the heterogeneity of contemporary families. Practitioners working with adults with a history of parental childhood abuse who are caregiving for their perpetrator are encouraged to employ a trauma-informed approach to maximize the caregivers’ health and well-being.

